# From Waste to Resource: Valorization of Lignocellulosic Agri-Food Residues through Engineered Hydrochar and Biochar for Environmental and Clean Energy Applications—A Comprehensive Review

**DOI:** 10.3390/foods12193646

**Published:** 2023-10-02

**Authors:** Silvia Escudero-Curiel, Alba Giráldez, Marta Pazos, Ángeles Sanromán

**Affiliations:** CINTECX, Department of Chemical Engineering, Universidade de Vigo, Campus As Lagoas-Marcosende, 36310 Vigo, Spain; sescudero@uvigo.es (S.E.-C.); alba.giraldez.rodriguez@uvigo.es (A.G.); mcurras@uvigo.es (M.P.)

**Keywords:** agri-food residues, hydrochar, biochar, engineered char, adsorption, clean energy, catalyst, valorization, circular economy, zero waste

## Abstract

Agri-food residues or by-products have increased their contribution to the global tally of unsustainably generated waste. These residues, characterized by their inherent physicochemical properties and rich in lignocellulosic composition, are progressively being recognized as valuable products that align with the principles of zero waste and circular economy advocated for by different government entities. Consequently, they are utilized as raw materials in other industrial sectors, such as the notable case of environmental remediation. This review highlights the substantial potential of thermochemical valorized agri-food residues, transformed into biochar and hydrochar, as versatile adsorbents in wastewater treatment and as promising alternatives in various environmental and energy-related applications. These materials, with their enhanced properties achieved through tailored engineering techniques, offer competent solutions with cost-effective and satisfactory results in applications in various environmental contexts such as removing pollutants from wastewater or green energy generation. This sustainable approach not only addresses environmental concerns but also paves the way for a more eco-friendly and resource-efficient future, making it an exciting prospect for diverse applications.

## 1. Introduction

**Figure 1:**
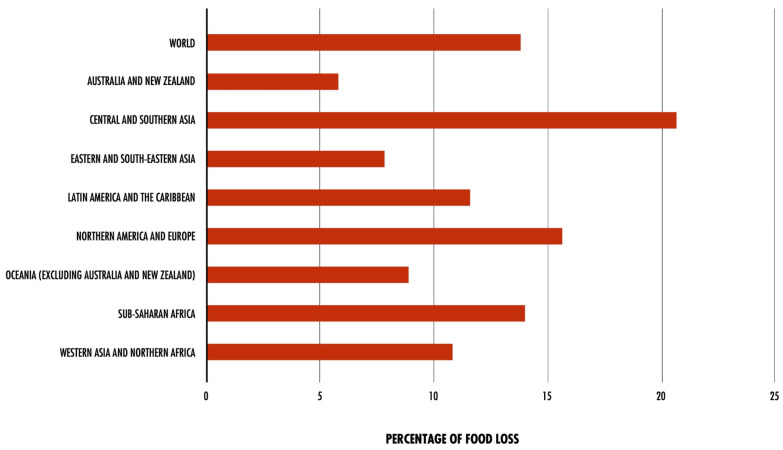
Percentage of food lost from harvest to distribution during 2016, globally and by region. It is important to clarify that this percentage refers to the physical amount lost for different groups divided by the amount produced. Figure source: FAO. 2019. The State of Food and Agriculture 2019. Moving forward on food loss and waste reduction [2].

Ensuring the sustainability of the human race requires addressing the multitude of pressing global challenges that persist today as a top priority. These challenges include fossil fuel depletion, air pollution, greenhouse gas emissions (GHG), and food security, as well as food waste generation and disposal. In recent years, the continuous growth of the global population has forced industrialization and modernization of the food and agro-industrial sectors to meet the high demand. This has resulted in increased pressure on management and waste disposal, which amplifies the urgency of the issue [1]. Figure 1 illustrates the variation in food wastage, from harvest to distribution, across different global regions in 2016, as mentioned above.

**Figure 2:**
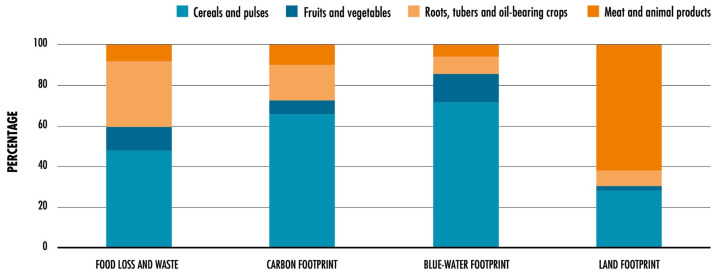
Relative contribution in percentage of the main food groups to overall food loss and waste, as well as to carbon, blue-water, and land footprints, by stacking these residues on bars. Note that these data refer to 2015 and that this figure was obtained from: FAO. 2019. The State of Food and Agriculture 2019. Moving forward on food loss and waste reduction [2].

This increased pressure leads to an escalation in GHG, alongside water scarcity and pollution, soil desertification, and biodiversity loss. These repercussions collectively contribute to climate change, highlighting the adverse effects of humanity’s growing ecological footprint [3]. Within this growing ecological footprint, it is important to focus on the carbon footprint (CFP), which is ‘the carbon dioxide in tons equivalent to the GHG produced by anthropogenic activities’ [4]. The CFP is highly contributed to by the agri-food chain, making it essential to reduce agri-food residues and manage its sustainably as a crucial step to mitigate climate change. Figure 2 shows the contribution of major food waste to carbon, blue water, and land footprints.

**Figure 3:**
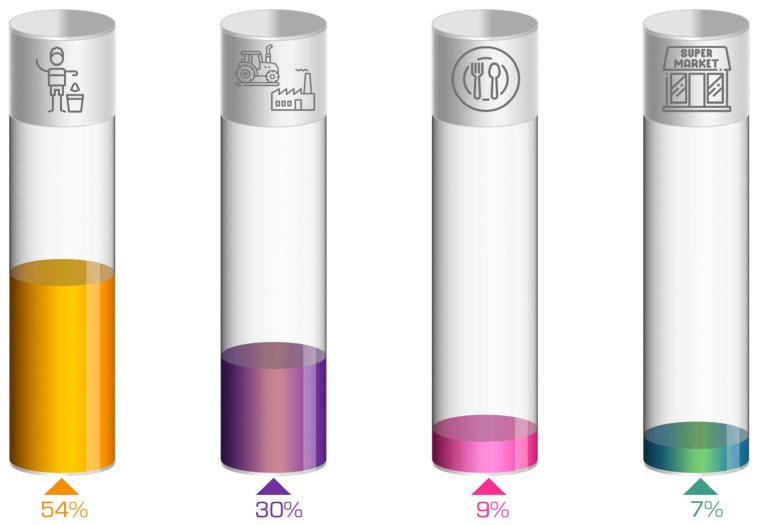
Percentage of food waste at different sectors of the supply chain in the EU. From left to right: households, primary sector and manufacturing of food products and beverages, restaurant and food service, retail and distribution. Data Source: Eurostat Statistic Explained. Food Waste and Food Waste Prevention-Estimates 2021 [7].

Food manufacturing residues or by-products and agri-food residues or by-products all pertain to the same type of residue and are characterized by its complex nature, as it can be generated at any part of the agri-food chain production process due to overproduction or processing and distribution problems, or directly in the agricultural sector [5]. These agri-food residues represent a missed opportunity to provide sustenance for a substantial portion of the world’s population. Today, it is estimated that the losses resulting from food manufacturing would feed 2 billion people, and it is known that 700 million people are currently experiencing hunger [6]. The FAO’s 2019 Assessment of Global Food Losses and Waste [2] estimates that each year about 14% of the world’s food production continues to become waste after it is harvested and before it reaches the stores. This is not only an economic and food security loss, but also a waste of all the natural resources used in the cultivating, processing, packaging, transportation, and marketing of food. The European Union (EU) currently generates 89 million tons of food waste per year [3]. The household sector (53.44%) and the primary production and manufacturing sectors (30.53%) are responsible for 84% of this amount (Figure 3) [7].

Traditional waste management, including methods such as anaerobic digestion, incineration, composting, utilization of fertilizer or animal feed, and landfilling, cause groundwater pollution through both leaching and infiltration, and air pollution through the emission of GHG, as well as dioxins and ash [8]. The CFP generated by this waste is estimated at 3.3 Gt of carbon dioxide equivalent per year [9]. Hence, it is vital to develop practices that help to reduce this waste in order to sustain the global economy, combat climate change, and improve human health. For this reason, various strategies are currently under research and are directed toward the actual transformation of waste into a product for a new purpose, reusing food waste, and thus, focusing on valorization. As a result, the recovery of agri-food residues has recently evolved from a trendy green movement to an urgent necessity [5].

Examples of such agri-food residues include mainly vegetable materials such as peels, straws or stems, leaves, seeds, and pomace [5]. As previously mentioned, agri-food residues are an abundant and readily available biomass feedstock. They contain a substantial amount of organic matter predominantly consisting of varying proportions of cellulose, hemicellulose, and lignin [3]. This composition makes them an exceptional and cost-effective source of carbon to produce biochar and hydrochar.

**Figure 4:**
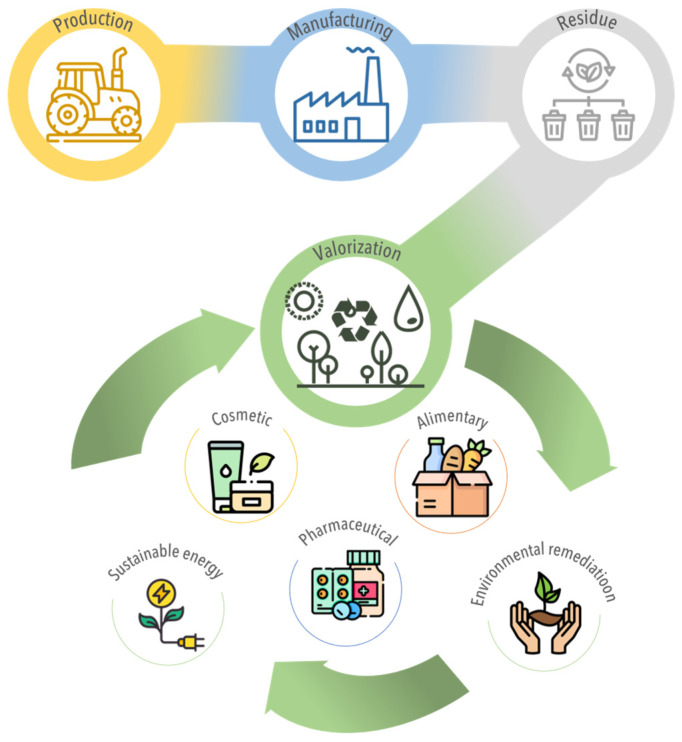
Circular economy cycle for agri-food residues illustrating the diverse industries benefiting from the valorization process and utilization of the obtained compounds.

Hence, these residues, often regarded as waste products with limited economic value, offer highly appealing feedstock options for thermo-conversion processes. The reuse of agri-food residues through thermochemical conversion represents an opportunity to embrace the principles of Zero Waste and to transit to a circular economy model of production and consumption. Circular economy involves sharing, leasing, reusing, repairing, renewing, and recycling existing materials and products for as long as possible, thereby minimizing waste generation and resource depletion. Nevertheless, the inherent complexity of agri-food residues, characterized by their diverse composition, distribution, and short lifespan, poses challenges to its valorization [10]. In this regard, effective management strategies, as well as strong efforts by individuals, organizations, and governments, are therefore essential to meet societal and environmental demands. Some authorities have already started to take initiatives, such as the EU, which has included food waste recovery as a priority in the Action Plan for the European Circular Economy Strategy or the European Green Deal initiative [11,12]. The United Nations has also addressed the issue in the Sustainable Development Goal to ‘ensure sustainable consumption and production patterns’, which proposes a target date of 2030 for ‘having global per capita food waste at the retail and consumer level and reducing food waste along production and supply chains’ [13]. In order to make these plans and goals economically viable, the variolization of agri-food residues can be achieved through the production of useful compounds for different industrial sectors, such as cosmetics, pharmaceuticals, biorefineries, or environmental remediation (Figure 4) [5].

This review provides an overview of emerging research strategies for valorizing lignocellulosic agri-food residues and achieving socio-economic and environmental benefits. To accomplish this, various types of vegetable waste from food manufacturing and agro-industrial sectors are analyzed. The review examines different thermal treatments employed to convert these residues into value-added products. The primary focus is on thermochemical conversion methods for producing biochar and hydrochar, with an emphasis on environmental applications, particularly in wastewater pollutant adsorption. Additionally, the review examines different engineering strategies for enhancing the adsorbent capacity and improving catalytic activities, as well as clean energy production. Furthermore, the review presents an evaluation of these strategies and offers insights into future perspectives.

## 2. Lignocellulosic Agri-Food Residues

**Figure 5:**
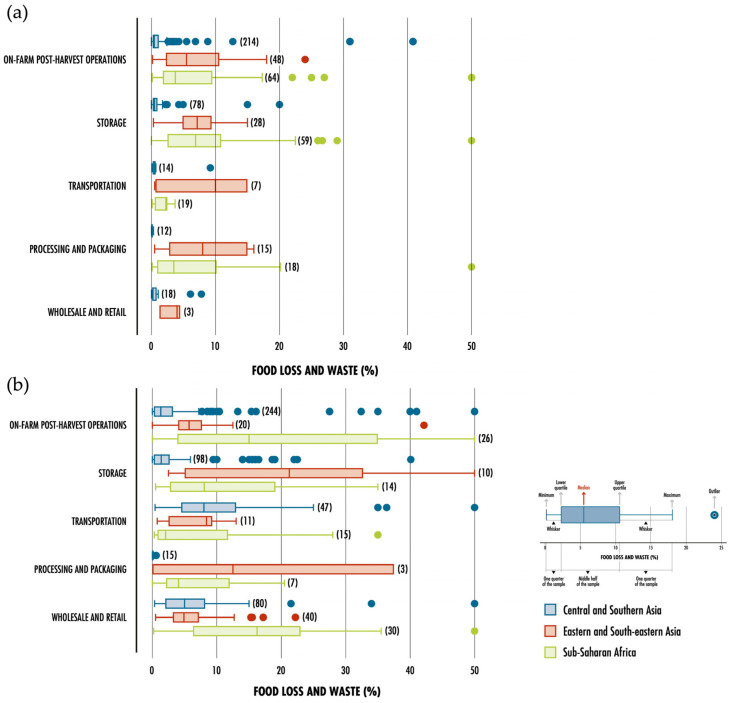
The percentage of agri-food losses, categorized as cereal and legume waste (**a**) and fruit and vegetable waste (**b**), during the period from 2000 to 2017 by region and stage of the supply chain are presented in the figure. The number of observations is shown in parentheses. Data source: FAO. 2019. The State of Food and Agriculture 2019. Moving forward on food loss and waste reduction [2].

The management of agri-food residues is considered a serious global environmental problem, particularly in developing countries. The main issues that these countries are facing are the lack of infrastructure in the transport and storage network for these products, and the lack of scientific knowledge and specific legislation for their long-term management [5,14]. Agri-food residues can be generated at the five stages of the food manufacturing value chain. At the production stage, losses occur mainly during the harvesting of cereals, fruits, and vegetables due to non-standard measurements or defects, mechanical or meteorological damage, and harvesting at inappropriate times [5]. Losses also occur during the transport, handling, and storage of products, usually due to degradation by micro-organisms, disease, handling, and even inefficient transport networks [5]. Processing and packaging are the stages with the lowest losses [5]. Figure 5 displays the aforementioned losses of these types of foods associated with each stage of the supply chain, as well as by region.

Hence, depending on the source of agri-food waste, different methods are required to pre-process the desired feedstock for recovery. If the residues are intermixed with other waste materials, they need to be manually sorted and separated by biomass of interest, or automated systems can be employed, which use various techniques depending on the characteristics of the target biomass. Such techniques include screening, filtration, centrifugation, or magnetic separation [15,16]. This is done to avoid impurities during the biomass recovery process [17]. After washing the selected biomass to eliminate any residue, contaminants, or dirt, excess moisture is then removed through drying. If required, the remaining material is shredded or ground to reduce its size [17].

### 2.1. Characteristics and Composition

Agri-food residues encompass an array of organic materials, including peels, seeds, shells, pomace, husks, straws, barks, and leaves [18]. These residues possess inherent worth attributed to their abundance of bioactive compounds such as phenols, peptides, carotenoids, and anthocyanins. Additionally, they contain valuable compounds such as polyphenols, alkaloids, and terpenes, along with polysaccharides, enzymes, lipids, minerals, vitamins, amino acids, and various other constituents.

In addition, these agri-food wastes may contain harmful substances such as pesticides, pathogens, or heavy metals [19]. This poses a risk to the environment and human health. Therefore, pre-treatment stages are necessary before the biomass can be utilized. The waste can be used for various purposes due to its specific properties [20,21]. One potential application of these substances is as adsorbents, because, as mentioned above, it has been reported that they can have toxic elements attached to them. Consequently, these substances can adsorb these elements and thus be used to remove environmental toxins by adsorption. For instance, a study conducted by Alslaibi et al. [17] illustrates this capability. Another example is that they serve as a versatile culture media for promoting microorganism growth, leading to the production of valuable chemicals such as aroma compounds, pigments, and essential oils. Additionally, these residues contain bioactive compounds, such as phenolic compounds, peptides, carotenoids, and dietary fibers, which exhibit a range of beneficial properties including antibacterial, antifungal, anti-inflammatory, immunomodulatory, and antioxidant activities. As a result, these residues hold significant potential as food supplements and additives in various sectors, including biomedicine, cosmetics, food, and pharmaceutical industries [5]. An example of circular economy and valorization of agri-food residues through different industries is shown in Figure 3.

**Figure 6:**
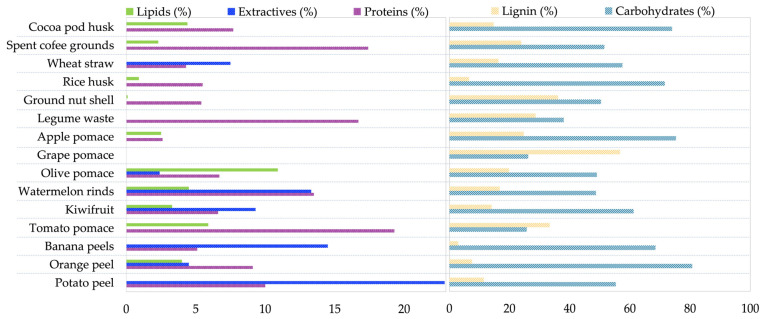
Composition of some examples of agri-food residues. Data source from Ebikade et al. [10].

In the present review, the attention is focused on lignocellulosic agri-food residues. These residues are great candidates or precursors as resources for use in applications such as green alternatives in environmental remediation, due to their high availability and their contribution to reducing the CFP [22]. These residues are also characterized by their chemical composition of carbon, hydrogen, oxygen, nitrogen, and traces of sulfur and chlorine [23]. Notwithstanding, different raw materials can be converted in different products depending on their elemental composition, so that, depending on the application, one or another type of waste is selected and one or another technique is used for its transformation [22]. In order to store these lignocellulosic agri-food residues and to be able to use them in some processes, it is essential to dry them, as some of these wastes are susceptible to microbial degradation due to moisture [24]. Figure 6 and Table 1 show some examples of lignocellulosic agri-food residues and their compositions.

### 2.2. Thermal Treatments for Valorization

Some of the most commonly used valorization processes are recycling, composting, and energy recovery, but they are unable to convert even 50% of the waste into value-added products such as energy, chemicals, and fuels [36,37]. Thermal treatments (such as pyrolysis, hydrothermal carbonization (HTC), hydrothermal liquefaction, and gasification), or biological processes (such as fermentation and combined chemoenzymatic) are some of the most demanded complex valorization techniques from a circular economy and carbon footprint reduction point of view [14,38].

**Figure 7:**
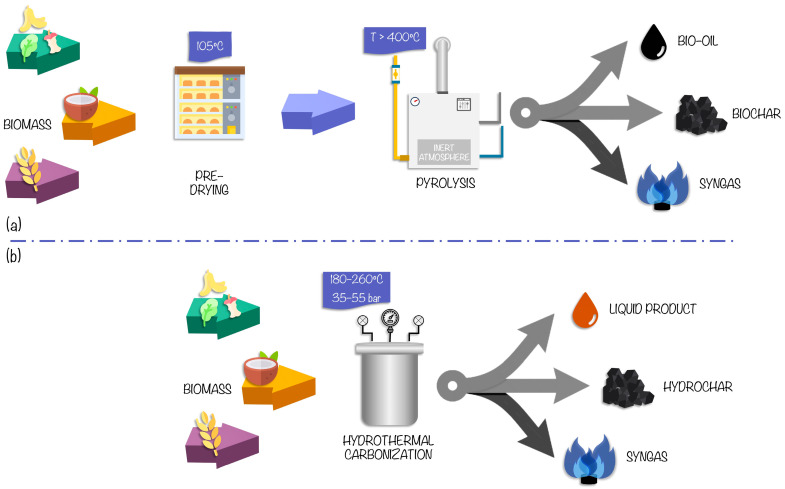
Diagram of (**a**) pyrolysis and (**b**) HTC processes.

In Figure 7, schematizes the waste valorization process using pyrolysis and HTC, which are the techniques we will focus on in this review. The general principles of these techniques are presented in the following.

Pyrolysis is the process with the shortest duration and the smallest infrastructure footprint compared to the other conversions [8,39]. The process consists of decomposing agri-food residues rich in lignocellulosic components in an oven of inert atmosphere at temperatures above 400 °C [22]. Depending on the heating rate, temperature, pressure, and residence time, it can be classified as slow or fast pyrolysis [40]. The obtained material is usually biochar (with >80% carbon), bio-oil, and, in smaller quantities, syngas. Typically, as the temperature increases during the process, the syngas production increases, and the biochar yield decreases because the interactions between the lignocellulosic components depend on the reaction temperature [40]. Hence, depending on the temperature, residence time and the material used, biochar is produced for some applications or others [41]. Biochar is mainly characterized by being a porous solid material rich in carbon, with a high specific surface area and a high presence of functional groups [42]. The main disadvantage of pyrolysis is that it requires complete drying of the feedstock, hindering its scaling-up for industrial applications [10].

In order to avoid this pre-drying stage of the biomass, hydrothermal techniques are considered efficient and promising technologies for the carbonization of wet biomass. These techniques are characterized by the heating and pressurization in autoclave reactors of agro-industrial waste in the presence of water [43]. Depending on the composition of the waste to be treated and the material to be obtained, there is a hydrothermal liquefaction process and an HTC process, which differs mainly in the temperature range and operating pressure of the process. The HTC process takes place at temperatures between 180 and 260 °C and pressures between 35 and 55 bar [10]. Upon completion of the hydrolysis, dehydration, decarboxylation, aromatization, and recondensation reactions take place, and a solid product, hydrochar (with 55–70% carbon), and a liquid product are obtained [44]. The hydrochar has a smaller surface area, while the liquid product has limited applications due to its high concentration of acids and phenols [10,44]. In addition, a gaseous product is also obtained, which is carbon dioxide and smaller quantities of hydrocarbons. Hydrochar can be used for different purposes, depending on several factors during its production. These factors include temperature, residence time, solid-liquid ratio, and feedstock [45]. Table 2 shows the elemental composition of some examples of biochar and hydrochar produced from agri-food residues.

There are certain disadvantages associated with valorization using these treatments, such as the difficulty of scaling up these processes to an industrial level, since the obtained products are usually in powder form, which makes them difficult to handle. In addition, it is necessary to carry out energy efficiency studies in order to optimize the valorization process of these low-cost feedstocks, so that the obtained product is more profitable than commercial products.

### 2.3. Biochar and Hydrochar as Adsorbents

Both biochar and hydrochar are characterized as carbonaceous materials with a broad range of applications, due to their profitable physicochemical properties, such as in the field of catalysts and clean energy generation, as well as in the field of fuels. However, their use in the adsorption process stands out among the different applications [42,47]. Biochars are known for their impressive surface area and negative surface net charge, which make them highly effective sorbents for cations, while exhibiting lower efficiency for anions and oxyanions. Although hydrochars may not have as large a specific surface area as biochars, they do have a significant abundance of functional groups on their surface [48]. The adsorption technique has proven to be highly suitable for environmental remediation using chars derived from agri-food residues, particularly in the removal of pollutants from wastewater that the current WWTPs are not able to remove [49,50,51,52,53].

**Figure 8:**
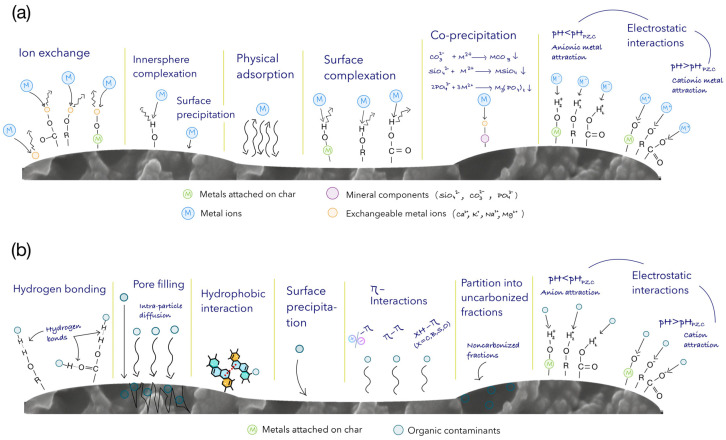
Summary of typical mechanisms for adsorption of (**a**) heavy metals and (**b**) organic pollutants onto biochar and hydrochar.

In addition, biochar is also capable of carbon sequestration, such as the removal of heavy metals and other air pollutants [22]. Several authors determined that different mechanisms contribute to adsorbent affinity for the target pollutant in aqueous media. A scheme of the adsorption mechanisms onto biochar for pollutants of diverse nature is shown in Figure 8. Some examples of the utilization of biochar and hydrochar as adsorbents derived from agricultural and food waste products and their corresponding characterization data are delineated below in Table 3.

The direct application of hydrochar and biochar for the removal of pollutants in the aquatic environment has been widely studied in the last years [47,54,55,56]. Thus, it has been determined that biochar and hydrochar present several technical barriers when used as adsorbents. Among these, the main one is the difficulty of obtaining a constant and optimized adsorption performance because it is affected by the raw material used and the production conditions, since, depending on these two elements, the surface area, porosity, and surface chemistry of the biochar or hydrochar will be obtained, which, in turn, is directly related to the adsorption capacity of these products [57,58]. Other important barriers that they present are related to the regeneration of these adsorbents, because it can damage the adsorption properties of the material and limit its reuse, as well as related to the inadequate selectivity or cost-effectiveness of using these adsorbents on a large scale, due to the complex production processes involved in using large amounts of raw material to produce these products at an industrial level [58,59].

However, literature reviews have focused on the detailed study of the adaptation of their structures in order to improve their physicochemical properties for adsorption purposes, and none have focused on the use and type of agri-food waste as raw material. Therefore, in the following sections, the engineered chars obtained in single-step or multi-step pathways from agri-food residues will be presented and discussed. Furthermore, the adsorption mechanisms and the surface properties will be highlighted and commented on. At the end, new applications for environmental purposes and future perspectives will be disclosed.

**Table 3:** Hydrochars and biochars from agri-food residues for the adsorption of organic and inorganic pollutants present in wastewater.

Waste	Biochar/Hydrochar	Medium	T (°C)	Time (h)	BET Surface Area (m^2^/g)	Pollutant	Adsorption Capacity (mg/g)	Reference
Olive waste	Hydrochar	Water	250	1.5	4.5	Methylene blue	15.1	[60]
						Congo red	11.78	
Olive oil production waste	Hydrochar	Water	220	2.5	-	Fluoxetine	4.6	[61]
						Cefazolin	0.4	
Orange peel	Hydrochar	Water	180	1	46.16	Methylene blue	66.23	[62]
Grape skin	Hydrochar	Water	180	1	34.08	Methylene blue	36.63	[62]
Potato peels	Hydrochar	Water	200	25	611.84	Congo red	147	[63]
Pomegranate pulp waste	Hydrochar	Water	220	12	3.52	Rhodamine B	121.95	[64]
Orange peel	Biochar	-	700	6	-	Cd	31.50	[65]
Rice husk	Biochar	H_3_PO_4_ solution	700	2	372.21	Tetracycline	552	[66]
Tomato waste	Biochar	-	600	1	1093	Methylene blue	385	[67]
Corncob	Biochar	-	400	1	-	NH4^+^	3.93	[68]

## 3. Engineered Biochar and Hydrochar

**Figure 9:**
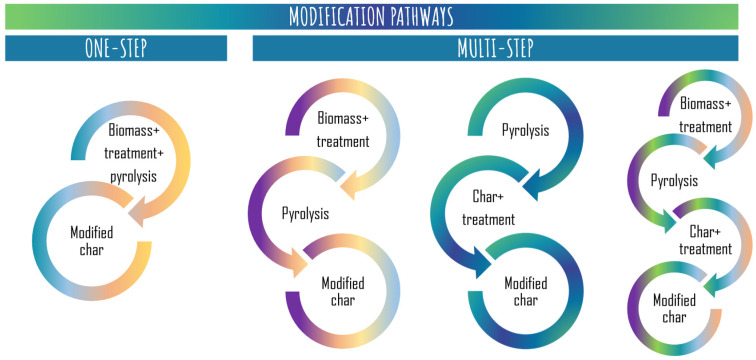
Pathways for modifying biochar and hydrochar.

The remarkable properties and impressive performance of biochars and hydrochars also provide the advantage of easy customization. Its complex and rich-in-functional-groups surface offers an exceptional platform for implementing alterations on it [57]. Novel approaches have been developed to modify biochars and hydrochars and to boost their adsorption capacity for both inorganic and organic pollutants in water, which expands the spectrum of chemical contaminants that can be effectively eliminated [69]. Surface engineering of these materials involves tailoring their physical and chemical characteristics to suit specific applications. This approach offers a simplified design and the adaptability to meet various industrial requirements. The choice of modification techniques and precursor characteristics directly influence the resulting surface functionalities [70]. The comprehensive modification of biochar and hydrochar aims to enhance not only their adsorption capacity, but also address practical limitations, such as selectivity, chemical and thermal stability, mechanical strength, and the presence of unwanted organic compounds in treated water, such as dissolved organic carbon from agro-industrial waste [59]. Hence, the range of techniques accessible for its modification aim to enhance the efficacy of these valorized products by modifying their physical and chemical properties, including surface area and functionality. These modification techniques involve employing both single-step and multi-step methodologies or pathways (Figure 9). In the single-step pathway, carbonization and modification take place simultaneously. On the other hand, the multi-step pathway entails either pre-treating the feedstock prior to pyrolysis or post-treating the carbonized product after pyrolysis, or even a combination of both approaches [58].

**Figure 10:**
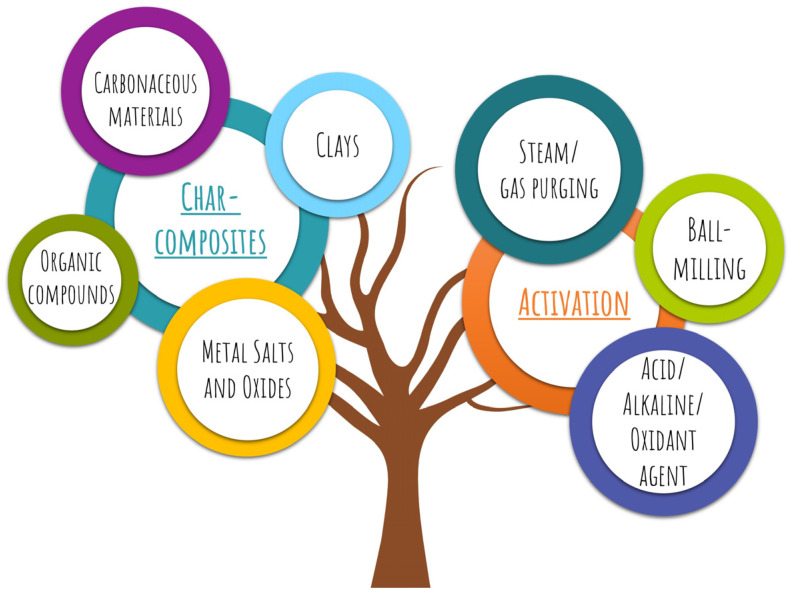
Chemical and physical methods of biochar and hydrochar modification.

Different ways, including chemical or physical modifications, may include treatments with oxidizing agents, gas purging, or char-composites, which involve incorporating different materials into the char surface composition. Engineering or tailoring the chars may lead to the valorization process incurring supplementary expenses; nevertheless, the cost/benefit ratio would exceed such costs. The following lines provide an overview of some key modifications that are also summarized in Figure 10.

### 3.1. Activation Strategies

Multiple activation processes can be applied to synthesized materials in order to enhance and modify their properties. In the following sections, a description of these processes is presented, and the more noteworthy studies reporting these types of activations are summarized in Table 4.

#### 3.1.1. Chemical Activation

Acidic, alkaline, and oxidizing agents are commonly utilized for the activation of adsorbents, aiming to enhance their surface properties, such as specific surface area and smoothness. It is common practice to utilize these agents in combination with one another, or in conjunction with other modification methods [85].

##### Acid Agents

Acid treatment is an oxidative process that involves applying acids to the surface of chars. Traditionally, strong acids, such as HCl, H_2_SO_4_, HNO_3_, and H_3_PO_4_, have been utilized. However, greener options, such as acetic, citric, or oxalic acid, have collected increasing interest in recent studies [52,86]. The primary objective of acid modification is to eliminate impurities, such as metals, while simultaneously introducing oxygen-rich active functional groups. This includes functional groups such as carboxyl, phenolic, hydroxyl, ester, and carbonyl, resulting in a raise in surface polarity and hydrophilicity [87]. Thus, acid treatments enhance the ability of chars to adsorb positively charged pollutants. Additionally, the acid-induced modification stimulates the development of interconnected micropores, ultimately leading to a notable increase in the overall surface area [52].

Hayoun et al. [52] produced loquat cores hydrochar in one-pot using three acids: two strong acids, namely H_3_PO_4_ and HCl, and one weak acid, citric acid. The researchers obtained a hydrochar with an increase in oxygenated groups on the surface and a specific surface area 20-fold higher than that obtained for hydrochar, without any modification. Besides, the adsorption capacity went from an average removal rate of 8.96% for diclofenac, prednisolone, and antipyrine to an average of 84.11%. He et al. [71] effectively produced engineered hydrochar from shrimp shells by employing a series of treatments. This involved pre-oxidation treatment with NaOH to facilitate deprotonation and deacetylation, following HTC, and post-oxidation treatment with acetic acid, which resulted in the acid etching of calcium carbonate. Acetic acid treatment produced a hydrochar with substantially amplified BET surface area, measuring almost six-times greater than the untreated and with an increased adsorption capacity of 755.08 mg/g for methyl orange. A significant increase in carboxyl and phenolic groups on the surface was reported in an HNO_3_-treated hydrochar derived from orange peel [72]. This HNO_3_-treated hydrochar exhibited a maximum adsorption capacity for methylene blue of 115 mg/g, whereas the untreated orange peel hydrochar achieved only 60 mg/g. Yu et al. [88] pyrolyzed shrimp shells at different temperatures (400–800 °C) and underwent post-oxidation with HCl. High pyrolysis temperature (800 °C) and post-oxidation enhanced biochar properties, resulting in high carbon content (84 wt%) and BET surface area (594 m^2^/g). These characteristics improved adsorption capacity, allowing for the removal of 75% of 2,4-dichlorophenol from wastewater.

##### Alkaline Agents

Alkaline treatment increases adsorption capacity by promoting pore opening, increasing surface area, introducing oxygenated functional groups creating proton donating exchange sites, and enhancing adsorption capabilities [58]. Commonly used alkaline agents such as KOH and NaOH are effective in increasing the surface area and oxygen content on the surface of the chars, facilitating the chemical adsorption of target pollutants [69]. Treatment using KOH and NaOH agents not only increases the oxygen content and surface basicity, but also aids in the dissolution of ash and condensed organic matter, which, in turn, improves the interaction and capture of pollutants. Factors such as feedstock type, preparation methods, and the ratio of alkaline agent to char influence the modification outcomes [47]. Although KOH is widely utilized as a modifier, enabling the production of activated chars characterized by a well-developed porous structure and high specific surface area, it is known as a toxic-strong base. However, its toxicity level decreases during the chemical activation process as it transforms and recovers to carbonate (K_2_CO_3_), which is generally considered to be less toxic than KOH [89].

Abbaci et al. [73] studied the adsorption of azo anionic dye azorubine and cationic dye methylene blue by a KOH-modified potato peel biochar. The modification was performed in two steps: one-pot KOH second pyrolysis, followed by washing with HCl to eliminate excess chemical agents and residual inorganic elements. The authors observed a remarkable enhancement in the specific surface area, with the untreated biochar initially measuring 0.84 m^2^/g, and the KOH-modified biochar exhibiting a significantly increased value of 2394 m^2^/g. This leads to outstanding adsorption results for removing azorubine, 2521 mg/g, and methylene blue, 667 mg/g, proving the effectiveness of alkaline treatment. Other studies have also reported similar findings; Tsai et al. [90] studied the capacity of a KOH-modified rice husk biochar to adsorb malachite green, demonstrating significant improvements in adsorption capacity attributed to notable increases in BET surface area and the presence of oxygenated functional groups on the surface. Findings by Liu et al. [85] revealed a substantial impact of NaOH pre-activation on the properties of shrimp shell biochar when loading Si/Mn binary oxide. This pre-activation process proved advantageous in facilitating a smoother surface morphology and achieving a more homogeneous distribution of crystalline Si/Mn oxide particles, finally increasing the adsorption of Cu^2+^. Petrović et al. [74] performed a KOH post-treatment to a grape pomace hydrochar for the purpose of Pb^2+^ adsorption. The obtained results demonstrated that the application of KOH treatment significantly enhanced the sorption capacity of the hydrochar, increasing it from 27.8 to 137 mg/g. The alkaline modification was found to effectively increase the content of oxygen-containing functional groups on the hydrochar surface.

##### Oxidizing Agents

Oxidizing agents, when employed for modification, can significantly boost the richness of oxygenated functional groups on both biochars and hydrochars [91]. Oxidizing agents, such as H_2_O_2_, other peroxy compounds, or KMnO_4_, have been suggested as a viable alternative to acids or alkaline compounds for char activation, especially for heavy metal adsorption. These agents offer the advantage of being less expensive and cleaner products, while addressing the environmental issues associated with the disposal of traditional activation media [92]. For instance, in the case of H_2_O_2_ utilization, it undergoes decomposition into harmless by-products of H_2_O and O_2_, leaving no adverse residues within the adsorbent materials.

Gomravi et al. [75] conducted a pre-oxidation of apple pomace using KMnO_4_, followed by the production of biochar for the Cu^2+^, Cd^2+^, Zn^2+^, and Pb^2+^ removal, with maximum adsorption percentages of 99.72%, 99.28%, 99.18%, and 96.45%, respectively. The resulting KMnO_4_-oxidized biochar exhibited a significantly higher BET surface area, approximately five times greater than that of the biochar modified with NaOH. Furthermore, the KMnO_4_-oxidized biochar demonstrated a considerable increase in the presence of functional groups such as C=O and C-O on its surface. Other studies reported that a coffee waste-derived biochar, subjected to a post-treatment with a NaOH/H_2_O_2_ solution [76], validated significant adsorption capacity for radioactive Sr^2+^, with a maximum uptake of 12.71 mg/g. Surprisingly, the BET analysis did not indicate an increase in specific surface area. The authors suggested that the presence of sodium salt in the post-treated biochar may prevent the collapse of pore structures on the surface, despite exhibiting a slightly smaller average pore size compared to the pristine biochar. In another study, post-treating corn stalk hydrochar with a 5% H_2_O_2_ solution had a significantly positive effect on Cu^2+^ and Cd^2+^ sorption capacity [77]. H_2_O_2_-hydrochar showed a remarkable adsorption of Cu^2+^, presenting an increase of 234.19% compared to the untreated hydrochar. Similarly, an increase of 99.03% for Cd^2+^ adsorption was verified for H_2_O_2_-hydrochar. The H_2_O_2_-treatment resulted in superior concentrations of O-containing functional groups (—OH and —COOH) in H_2_O_2_-hydrochar, thereby enhancing the adsorption capacities of Cu^2+^ and Cd^2+^. Surface complexation is presumed to be the leading sorption mechanism for hydrochar. Similar results were found by the same research group when removing atrazine [93].

#### 3.1.2. Physical Activation

Typically, physical modification techniques are relatively easy to apply and are cost-effective, although they may not be as potent as chemical modification methods. The physical modification process relies on the utilization of oxidizing agents such as carbon dioxide (CO_2_), steam, ammonia (NH_3_), oxygen, nitrogen, air, or even a mixture of them, without the need for chemical additives [58].

##### Steam and Gas Purging

Steam and gas purging modification are two-step processes that involve the pyrolysis of the feedstock followed by the gasification of biochar. On the one side, during the steam modification process, the oxygen molecules in water are exchanged with the active sites on the carbon surface of biochar. This exchange results in the formation of surface hydrogen complexes as the hydrogen gas generated from the loss of oxygen in water reacts with carbon on the biochar surface [58]. On one aspect, steam modification facilitates devolatilization, which indicates the release of volatile components from biochar and endorses the formation of crystalline carbon structures within the biochar [94]. This helps to enhance the stability and durability of the modified biochar. On the other aspect, steam modification assists the removal of the trapped products of incomplete combustion that may have formed during the initial pyrolysis process, thus increasing the surface area of the biochar and improving its structural characteristics [95]. Hence, steam modification can enhance the adsorption capacity of biochar, allowing it to effectively capture and retain pollutants or other target substances from various media [96,97]. On the other side, gas purging typically uses CO_2_ or NH_3_ gas. This technique enhances the surface area and structure of biochar. Furthermore, the process of gas purging effectively accelerates the thermal decomposition of carbonaceous substances and intensifies the aromatic nature of the biochar [94]. Through a chemical reaction, CO_2_ interacts with the carbon present in biochar, leading to the formation of CO (termed hot corrosion) and contributing to the development of a highly intricate microporous configuration, also resulting in an augmented surface area of the biochar [98]. NH_3_ gas modification introduces nitrogen-containing groups on the biochar surface, which cause an increase in alkalinity, enhancing its surface chemistry and providing additional active sites for adsorption [47]. This activation technique raises the development of amides, lactams, and imides functional groups through dehydration, decarboxylation, or decarbonylation reactions [94].

Bouhadjra et al. [78] conducted a physical post-activation to enhance the properties of potato peels biochar. The biochar was subjected to a temperature of 800 °C for 30 min under a steam flow rate of 10 g/min. The results revealed the presence of highly organized nanotubes within the biochar structure, creating a nano-porous framework that facilitates the adsorption and nucleation of dyes on the biochar. Remarkably, the experimental maximum capacity of the potato peel biochar for removing Cibacron blue dye reached an impressive 94%, significantly surpassing the capacity of the pristine potato peel (270.3 mg/g compared to 100 mg/g, respectively). This substantial improvement in adsorption capacity can be attributed to the reduction in -OH and C-O bonds, leading to an increase in aromaticity with more pronounced C=C skeletal vibration of the aromatic rings and the presence of C—O—C lactone structures. Román et al. [79] modified hydrochars from walnut shell, sunflower stem, and olive stone with air and CO_2_. Air-modified olive stone hydrochar exhibited high adsorption capacity for fluoxetine (44.1 mg/g), while CO_2_-modified hydrochar was effective for removing nicotinic acid (91.9 mg/g). Surface acidity played a main role in fluoxetine adsorption and nicotinic acid removal, being favorable in fluoxetine adsorption, but having an adverse effect on nicotinic acid removal. In a study conducted by Lian et al. [80], an innovative approach involving NH_3_ post-treatment was utilized to synthesize N-doped biochar from corn straw. A specific variant, NBC800-3 sample (800 °C in NH_3_, 3 h), showed a remarkable enhancement in dye adsorption capacity, 292 mg/g for acid orange 7 (anionic dye) and 436 mg/g for methyl blue (cationic dye), when compared to its pristine equivalent. Moreover, the BET surface area of this particular sample raised steeply from a modest 72.7 m^2^/g to 418.7 m^2^/g. Furthermore, NH_3_ activation demonstrated a substantial augmentation in the nitrogen content of the biochars, surpassing a twenty-fold increase. The dominant constituents were pyridine-like and pyrrolic-like nitrogen, constituting over 50% of the atomic composition within the N-doped biochars. Another research effort, conducted by Yu et al. [81], focused on exploring the potential of crop straw by subjecting it to NH_3_ post-treatment. The resulting N-doped biochar exhibited a graphitic-N percentage of 46.4% and presented a surface area of 418.7 m^2^/g. Consequently, this N-doped biochar exhibited elevated adsorption capacities for Cu^2+^ and Cd^2+^ (1.63 mmol/g and 1.76 mmol/g respectively), which were four-fold higher than those observed in the original biochar.

##### Ball-Milling

Ball-milling is an effective and green mechanochemical process that uses intense mechanical energy to achieve a reduction in particle size of pristine chars, transforming them into nanoparticles. This facilitates chemical modification without the need for chemical reagents [99]. Recent investigations have unveiled the benefits of ball-milling treatment in enhancing the adsorption capacity and augmenting the presence of oxygen-containing functional groups in carbon-based materials, leading to enhanced performance in diverse environmental applications [100,101]. Significantly, the effectiveness of ball-milling technology has been exemplified in the customization of biochar attributes by amplifying the surface area, diminishing particle dimensions enriching oxygen-containing functional groups, and optimizing adsorption and catalytic efficacy [82,83,84,102,103]. Although the field of ball-milled biochar is still nascent, it presents both challenges and opportunities for further exploration [99]. The process of ball-milling modification involves generating kinetic energy through the movement of milling media and substrates. This energy can successfully disrupt chemical bonds, resulting in the fragmentation of solid materials, formation of electrochemical elements, cleavage of glycosidic bonds, and transfer of charges [103].

Xiang et al. [82] and Zhang et al. [83] prepared ball-milled biochar, derived from wheat stalk and crayfish shells, respectively, to remove the antibiotic tetracycline from water. Both studies reported a significant enhancement in the tetracycline adsorption capacity of the ball-milled biochars, exhibiting an increase of approximately 1.5 to 3 times compared to their pristine counterparts. Through ball-milling, it was observed that crayfish shell biochar underwent a transformation, resulting in the generation of ultrafine particles (reduced from 50.56 μm to 0.623 μm) and the formation of an interconnected pore network. This led to a remarkable increase in the BET surface area by 2.26 times and a substantial enhancement in the volume of micropores by 2.18 times. Similarly, wheat stalk biochar exhibited significant improvements with an 8.5-fold increase in the volume of mesopores and a 4-fold increase in the BET surface area compared to its pristine state. Qi et al. [84] conducted an adsorption experiment to investigate the performance of pristine and ball-milled wheat straw biochar in adsorbing aromatic VOCs, namely benzene, o-xylene, m-xylene, and p-xylene. Remarkably, the ball-milled biochar exhibited significantly higher adsorption capacities, increasing 1.96–3.97 higher than those of the original wheat straw biochar. This improvement can be attributed to the effective enhancement of the biochar through ball-milling of the BET specific surface area, approximately 14.7 times higher than the pristine biochar, and total pore volume, ranging from 0.0069 to 0.0879 cm^3^/g. Studies have suggested that ball-milling can enhance BET surface area in two ways: by reducing particle size to increase the external surface area and by opening internal pores to increase the internal surface area [104].

Limited research has been conducted on the utilization of ball-milled hydrochar for adsorption purposes. As a result, there exists an opportunity to investigate this area and explore its potential applications.

### 3.2. Char-Composites

Bio-composites are advanced materials that combine two or more distinct substances to create a novel material with enhanced performance, surpassing that of its individual components [105]. Within these bio-composites, chars play a vital role as a porous framework that provides support for modifiers embedded within their matrix. This unique characteristic enhances the adsorption capacity, enabling the removal of various contaminants across a broad spectrum [106]. These innovative biomaterials, derived from biomass, have emerged as encouraging candidates for wastewater treatment. Their notable biodegradability, superior performance, and eco-conscious attributes have attracted significant interest and scrutiny in recent times [57].

#### 3.2.1. Metal Salts and Metal Oxides

To enhance the affinity of chars for pollutants of different natures, novel strategies have been developed by incorporating metal salts or metal oxides with positive charges onto the surface of the adsorbents [48]. Metals such as Fe, Mg, Al, and Mn have gained prominence in the modification process. The commonly utilized techniques include impregnating biochar with metal nitrate or chloride salt solutions and employing agents such as FeCl_3_, Fe^0^, Fe(NO_3_)_3_, and MgCl_2_ [69]. Subsequently, the impregnated adsorbent undergoes controlled heating under atmospheric conditions to eliminate nitrates or chlorides and efficiently transform the metal ions. This metal salt or metal oxide modification serves multifaceted purposes, including augmenting the adsorption capabilities for targeted pollutants and facilitating adsorbent recycling through magnetic properties [47].

Li et al. [107] investigated the production of a MnO-modified biochar for heavy metal removal by functionalizing rice husk biochar with MnCl_2_ and Na_2_CO_3_ before pyrolysis. The researchers examined the carbonization of biochars at different temperatures and found that those carbonized at 450 °C and 600 °C exhibited a single-phase MgO crystal structure and a higher abundance of highly active metal-oxygen bond functional groups compared to biochars carbonized at other temperatures. These functional groups played a crucial role in the precipitation and complexation mechanisms and immobilization of heavy metals, as is the case of the presence of highly active Mg-O bonds. Remarkably, the MnO-modified biochar achieved a maximum adsorption capacity of 191.80 mg/g for Cu^2+^. A Mn-Fe-modified rice straw-derived biochar [108] was produced by pre-treatment of the feedstock with FeSO_4_ and MnCl_2_ to remove atrazine, an agricultural herbicide, from aqueous solutions. The enrichment of O-containing functional groups (such as —OH, —C=C, and —C=O) and the incorporation of FeMnO_3_ into the biochar matrix resulted in a significant enhancement in atrazine removal. The maximum atrazine adsorption capacity of the modified biochar was 4.3 times greater than that of the untreated counterpart. The effective adsorption mechanisms of Fe/Mn-modified biochar primarily involved robust π—π interactions between atrazine molecules and the O-containing functional groups, as well as the presence of graphitic carbon and Fe/Mn-oxides on the biochar surface. Furthermore, the larger surface area and well-defined pore structure of the modified biochar facilitated enhanced adsorption and efficient pore filling of atrazine. Comparably, Ning et al. [48] fabricated a Fe/Mn-post-functionalized hydrochar by treating it with KMnO_4_ and FeCl_2_ to adsorb 17β-estradiol. The modified hydrochar exhibited exceptional adsorption capacity, with a value of 49.77 mg/g, and a significantly increased specific surface area of 167.17 m^2^/g. This improvement was approximately 375% higher compared to the pristine hydrochar and surpassed the adsorption capabilities of previously reported adsorbents up to the time of publication. In another study, conducted by Liu et al. [109], peanut shells were pre-treated with FeCl_3_ to produce zero valent iron magnetic biochar at various temperatures, aiming to enhance the capture efficiency of Cr^6+^ and trichloroethylene (TCE). A comparison between the Fe-biochar and without Fe, produced at the same pyrolysis temperature (800 °C), revealed that the specific surface area and total pore volume of Fe-biochar (FeBC800) were significantly lower. This was ascribed to the presence of iron oxide and zero-valent iron (ZVI) occupied the biochar pores. The adsorption capacity of FeBC_800_ for Cr^6+^ was attributed to the reductive properties of ZVI, which resulted in the reduction of some Cr^6+^ to Cr^3+^ during the adsorption process. Furthermore, FeBC_800_ demonstrated complete removal of TCE, indicating a simultaneous role of degradation facilitated by ZVI and adsorption. Comparably, Eltaweil et al. [110] carried out a study on the performance of magnetic corn straw-derived biochar supporting ZVI. They conclusively confirmed the oxidation of ZVI particles during the removal process, resulting in the reduction of malachite green dye. This reduction was facilitated by the electrons generated from the dissociation of Fe^0^, ultimately leading to the decolorization of the malachite green dye. Recently, Kohzadi et al. [111] synthesized wheat straw hydrochar using a one-pot method with FeCl_3_. The maximum adsorption capacity of the resulting hydrochar for rhodamine B dye reported was 80 mg/g. It was observed that the Fe-modified hydrochar exhibited greater porosity and surface roughness, and the BET surface area analysis revealed a significant increase from 9.4 m^2^/g to 52.74 m^2^/g, which is considerably higher for a hydrochar material. Additionally, the Fe-modified hydrochar displayed improved thermal stability. Khairy et al. [112] investigated the synthesis of iron nanoparticle-loaded hydrochar derived from pomegranate peels. The production of FeNPs@HC involved a one-pot HTC process in alkali medium, utilizing a black solution of iron nanoparticles as the HTC medium. The maximum sorption capacities for Cu^2+^ and methylene blue dye were found to be 95.24 mg/g and 278 mg/g, respectively. The BET surface area of FeNPs@HC was determined to be 24.37 m^2^/g. Analogously, Krishna et al. [113] synthesized an iron oxide magnetic hydrochar from coffee husk to assess its adsorption capacity from synthetic wastewater for methylene blue. It was prepared through co-precipitation of FeCl_3_.6H_2_O and FeSO_4_.7H_2_O with ammonia solution. The magnetic hydrochar exhibited an impressive monolayer adsorption capacity of 78 mg/g for methylene blue dye. Significantly, the magnetized particles produced exhibited a notable susceptibility to external magnetic fields, effectively resolving the challenge of separating powdered biochar from the aqueous matrix. This crucial advancement addresses the inherent drawback that renders it unappealing to end-users in various practical applications.

#### 3.2.2. Clays

Clay minerals are hydrous aluminum phyllosilicates, often containing additional elements such as iron, magnesium, alkali metals, alkaline earths, and other cations [40]. Clay materials find extensive applications in various industrial, engineering, and scientific fields, due to their low cost and advantageous physicochemical, structural, and mechanical properties such as particle size, superficial chemistry, particle nature, and surface area [106,114]. Clays possess interchangeable cations and anions such as Ca^2+^, Mg^2+^, H^+^, K^+^, Na^+^, NH_4_^+^, Cl^−^, PO_4_^3−^, and NO_3_^−^, permitting ion exchange without affecting the clay structure [40]. Previous studies have demonstrated the adsorption capabilities of clay particles, specifically on charged (cations and anions) and neutral pollutants in water, and primarily on the faces and limits of clay particles [40]. In this sense, low-cost chars provide an excellent porous framework for accommodating and distributing nanoparticles throughout its matrix. This attribute can be utilized to create an innovative engineered clay-biochar composite incorporating dispersed clay particles within its structure, achieving an enhanced adsorption capacity [57].

A broad range of clays, including both natural and synthetic varieties, are commonly employed in the preparation of composites. Within the natural clays applied are, among others, montmorillonite, hectorite, sepiolite, bentonite, vermiculite, beidellite, kaolinite, and chlorite. Additionally, synthetic clays, such as layered double hydroxides (LDH), are also incorporated in composite formulations [115]. Producing clay-biochar composites typically implies a pre-pyrolysis mixing of raw material with a suspension of the clay mineral [69].

A highly efficient adsorbent composed of rice husk-derived biochar supported with Mg-Fe-LDH was utilized for the effective elimination of several heavy metal ions, specifically Pb^2+^, Cu^2+^, Co^2+^, Ni^2+^, Zn^2+^, and Cd^2+^ [116]. Remarkably, the maximum adsorption capacity for Pb^2+^ reached 682 mg/g. The obtained results showcased the substantial abundance of diverse functional groups, including hydroxyl, carbonyl, and carboxyl groups. The BET surface area analysis significantly increased from 60 m^2^/g and 36 m^2^/g for the original LDH and rice husk, respectively, to 200 m^2^/g. Similarly, Jung et al. [117] synthesized a Mg–Al LDH-hydrochar composite from rice husk in a one-step, one-pot process for arsenate and phosphate removal. The clay-hydrochar composite exhibited a considerable quantity of exchangeable interlayer nitrate anions, providing stronger adsorbent–adsorbate electrostatic interactions, which stimulate intercalation of arsenate and phosphate into the interlayer region. This resulted in 97.8% and 94.6% removal of arsenate and phosphate, respectively. The author reported that intercalation, inner-sphere, and outer-sphere surface complexes were the main mechanism governing the adsorption process. In the research conducted by Wang et al. [118], an halloysite-biochar magnetic composite derived from coconut shell was prepared for the removal of Pb^2+^ in wastewater. The authors first produced halloysite-biochar through co-pyrolysis, and, subsequently, to impart magnetic properties to the composite, they ball-milled the composite with Fe_3_O_4_. The as-prepared adsorbent performed at 700 °C demonstrated exceptional Pb^2+^ adsorption capacity, with the highest recorded value of 833.33 mg/g. Additionally, the removal efficiency of Pb^2+^ remained consistently above 90%, even after undergoing four adsorption–desorption cycles. The reported BET surface area was 391.58 m^2^/g, 1.4 times higher than the pristine biochar, even though Fe_3_O_4_ form aggregates and causes a decrease in surface area and porosity compared with ball-milled biochar. Various mechanisms, including complexation, cation exchange, cation—π interaction, precipitation, pore filling, and electrostatic adsorption, have been suggested as the primary mechanisms for pollutant removal. The removal of the antibiotic norfloxacin was investigated by Li et al. [119] through an attapulgite-potato stem biochar composite (APB), and by Zhang et al. [120], using a montmorillonite-biochar composite (MT-BC) derived from wheat straw. The adsorption capacity of norfloxacin significantly increased by 48.9% in the case of APB, and by 241% in the case of MT-BC. The surface area of MT-BC was observed to increase from 20.1 to 112.6 m^2^/g, while the BET surface area of APB showed a slight decrease compared to the pristine potato biochar. However, the total pore volume and average pore width of APB were larger compared to the original biochar. APB mechanisms of adsorption may be mainly attributed to electrostatic attraction/repulsion, hydrogen bonding, and π—π electron–donor–acceptor. On the other hand, the enhancement of adsorption capacity of MT-BC composite was primarily attributed to hydrogen bonding between the norfloxacin- and oxygen-containing groups, as well as electrostatic attraction.

Few studies have been conducted utilizing agro-industrial residues, but not by-products derived from food manufacturing, specifically focusing on the utilization of clay-hydrochar composites.

#### 3.2.3. Carbon-Based Nanomaterials

Carbonaceous materials, such as carbon nanotubes (CNT), graphene, graphene oxide (GO), or reduced graphene oxide (rGO), have demonstrated remarkable adsorption capacities [121,122,123,124,125]. Despite their effectiveness in contaminant removal, their high cost and industrial limitations, such as difficulty in recovery after use, have hindered widespread application. As a solution, chars can be employed as supporting matrices for these nanomaterials, enabling the development of novel, competent, and recyclable adsorbents for treating wastewater [58]. As an example, the introduction of GO in the composite during pyrolysis leads to the formation of additional oxygen-containing functional groups, surpassing the quantity present in the unmodified biochar [126,127]. The inclusion of graphene greatly enhances the π—π interactions between the adsorbents and organic pollutants, resulting in significant improvements in adsorption capacity [128]. In addition, for heavy metals, graphene chemically binds with the oxygen-containing groups, further enhancing their adsorption capabilities [69]. Similarly, the hybrid structure of multi-walled CNT coated on the biochar enhances its surface areas, porosity, and thermal stability, outperforming the uncoated biochar [129].

In their study, Fan et al. [130] developed a corn cob-derived biochar composite by incorporating in situ biosynthesized iron oxide nanoparticles before pyrolysis and commercial CNTs post-pyrolysis to boost the removal of the antibiotic oxytetracycline from wastewater. The modified biochar exhibited a 1.89-fold increase in adsorption capacity compared to untreated biochar, reaching a theoretic maximum adsorption capacity of 72.59 mg/g. The composite demonstrated a BET surface area of 237.51 m^2^/g and showed mild magnetic properties. Importantly, the introduction of CNTs improved the surface area and pore structure of the composite. This composite effectively addressed the issue of agglomeration of iron oxide nanoparticles and CNTs, resulting in significantly enhanced adsorption capacity of the biochar. Yang et al. [131] utilized chestnut shells to create a composite of CNT and biochar for the removal of Pb^2+^. The resulting nanocomposite exhibited a high BET surface area of 231.60 m^2^/g and achieved a maximum adsorption capacity of 1641 mg/g. Remarkably, even after undergoing four cycles of adsorption and desorption experiments, the nanocomposite preserved an adsorption rate of 88.91%. This suggests that the nanocomposite possesses excellent stability and reusability. The authors indicated that the mechanisms responsible for Pb^2+^ removal by the nanocomposites were complexation with the oxygen-containing functional groups present on the sorbents, cation exchange, and electrostatic attraction with the surface. A two-step treatment of KOH activation followed by annealing was employed by Zhu et al. [132] to prepare a composite consisting of three-dimensional nano-Ni particles embedded in N-doped carbon nanotubes supported on biochar derived from sugarcane bagasse. The composite was evaluated as Cr^6+^ adsorbent and it exhibited a significantly high BET specific surface area of 604.62 m^2^/g. The activation treatment not only improved the doping efficiency of Ni and N atoms in the biochar, but also enhanced the yield and length of carbon nanotube growth on the biochar. Furthermore, the Cr^6+^ removal capacity of the composite was remarkably enhanced by more than five times after KOH activation.

Du et al. [133] prepared and characterized an innovative composite comprising Fe_3_O_4_ nanoparticles and graphene-biochar derived from rice straw, to enhance the adsorption capacity of crystal violet dye and recovery efficiency. The composite surface presented an increased surface area, improved thermal stability, and the presence of additional functional groups. These enhancements contributed to an augmentation in adsorption capacity compared to uncoated biochar, achieving a maximum adsorption capacity of 436.68 mg/g. Remarkably, the saturation magnetization (61.48 emu/g) enabled efficient recovery of the composite using a magnet.

A biochar-supported composite consisting of rGO was successfully prepared by Zhang et al. [134] through slow pyrolysis of corn straws pretreated with GO for removing atrazine and Pb^2+^. Analysis of the structure and morphology showed that GO nanosheets were coated onto the biochar surface primarily through π—π interactions. Importantly, even after annealing reduction, the GO nanosheets retained their original morphology. The resulting composite exhibited improved physicochemical properties on its surface, resulting in excellent adsorption capacities of 26.10 mg/g for Pb^2+^ ions and 67.55 mg/g for the herbicide atrazine.

To date, no studies have been identified that explore the utilization of hydrochar composites derived from food manufacturing residues or other agro-industrial processes containing these carbonaceous nanomaterials.

#### 3.2.4. Surfactants, Nitrogen-Rich Compounds, and Others

Customizing the surface of a carbon matrix with organic compounds is usually employed to introduce advantageous functional groups that could augment the adsorption capacity for target pollutants [135]. It is commonly accomplished by utilizing anionic or cationic surfactants and predominantly nitrogen-rich compounds such as urea, ammonia, or polymers such as polyethyleneimine (PEI) or chitosan [86,136,137,138,139]. Surface grafting with nitrogen-rich compounds introduces nitrogen-containing functional groups, including NH_2_, amides, pyridinic, pyrrolic, and graphitic nitrogen groups, onto the carbon surface [140]. This process enriches the electronic properties of the N-doped surface, leading to localized charge accumulation. This characteristic enables electron transfer reactions, facilitates strong complexation with pollutants, and also provides basic character to the carbon matrix [69,141]. This grafting process can be carried out either in a one-step, one-pot strategy [61], or in a two-step procedure involving the mixing of the carbonaceous matrix with the chosen compound [142].

A PEI-modified hydrochar from corn cobs, subjected to post-treatment with a crosslinker, glutaraldehyde (GTA) [142], exhibited exceptional capability in removing Cr^6+^ and Ni^2+^. The maximum adsorption capacities for Cr^6+^ and Ni^2+^ on the modified hydrochars were significantly enhanced, with values of 33.663 mg/g and 29.059 mg/g, respectively. These values represented an increase of 365% and 43.7% compared to the unmodified hydrochar. The presence of nitrogen-containing groups on the surface of the modified hydrochar indicated that these groups played a crucial role as the primary adsorption sites for both Cr^6+^ and Ni^2+^. Analogously, Qu et al. [143] synthesized a magnetic hydrochar with N-doping and PEI grafting through a post-HTC process assisted by microwave irradiation. Remarkably high uptakes of Cr^6+^ and BPA were achieved by the composite, reaching 205.37 mg/g and 180.79 mg/g, respectively. The presence of protonated amino groups, heterocyclic N structures, and Fe^+2^ ions significantly enhanced the uptake and reduction of Cr^6+^. As for BPA, its binding was greatly enhanced through the synergistic effects of pore filling, hydrogen bonding with N≡N groups formed through a diazo coupling reaction during the grafting process, and π—π interactions with graphitic N and Fe—NX structures, which played a crucial role. Analogously, a PEI-biochar was prepared for Cr^6+^ removal by Ma et al. [144]. The synthesis involved a post-pyrolysis step, which included alkali activation and PEI grafting using the crosslinker GTA, reporting a maximum adsorption capacity of PEI-alkali-biochar for Cr of about 435.7 mg/g. Similarly, Escudero-Curiel et al. [61] prepared two N-doped hydrochars derived from olive pomace, or “alperujo”, with urea and PEI in one-step, one-pot modification to remove a drug mixture of fluoxetine and cefazolin from aqueous media. In both instances, the formation of amide groups played a beneficial role in facilitating the adsorption process. Remarkably, PEI-hydrochar exhibited an exceptional maximum adsorption capacity of 983.84 mg/g for cefazolin, which represented an increment of approximately 330-fold compared to the untreated hydrochar. Fluoxetine achieved a significant adsorption capacity of 29.31 mg/g. The high adsorption capacity for cefazolin of PEI-hydrochar was attributed to strong electrostatic interactions between the positively charged active sites of PEI-hydrochar and the negatively charged carboxyl groups of cefazolin.

In a study conducted by El-Nemr et al. [136], the surface of biochar derived from watermelon peel was subjected to amination for the purpose of Cr^6+^ removal. This amination process was performed as a post-treatment, and two compounds, namely ammonium hydroxide and triethylenetetramine (TETA), were tested for grafting onto the biochar surface. The maximum adsorption capacities reported were 72.46, 123.46, and 333.33 mg/g for the unmodified biochar, ammonium hydroxide-grafted, and TETA-grafted, respectively. The amination process resulted in a reduction in pore size of the modified biochar, while simultaneously enhancing the surface area. Another example of surfactant used for producing biochar composites is sodium dodecyl sulfate, as is the case of the study conducted by Que et al. [145]. These authors conducted a study where they prepared an SDS-biochar composite from peanut shells. This composite showed an interesting maximum adsorption capacity of 503 mg/g for methylene blue due to a reasonable increase in the density of functional groups in the composite after the SDS grafting.

Afzal et al. [146] used hydrogel beads composed of a chitosan/biochar composite derived from grapefruit peels to remove the antibiotic ciprofloxacin from water. The chitosan/biochar composite, performed in a post-pyrolysis step, significantly boosted the adsorption capacity, increasing it by 11 times compared to the pristine biochar. The new —NH and —NH_2_ groups formed in the composite played a crucial role in this improvement due to the formation of hydrogen bonds between the —COOH group in ciprofloxacin and the -NH groups present in the chitosan/biochar hydrogel. Kamdod and Kumar [147] investigated the adsorption capacity of a chitosan/biochar composite derived from coconut shell for removing two cationic dyes, methylene blue and crystal violet, and one anionic dye, methyl orange. It was produced in a post-pyrolysis step using GTA as a crosslinker. These authors reported a notable enhancement in the adsorption of methyl orange adsorption, attributed to the anionic nature of the dye and its interaction with the N-containing groups in the composite. Due to the presence of amine functional groups (C–N and N–H) in the composite, the composite could adsorb anionic contaminants more effectively. Interestingly, there is scarce research existing on hydrochar/chitosan composites [138].

## 4. Novel Environmental Applications

Biochar and hydrochar derived from agri-food residues, as carbonaceous materials, have also been utilized as greener electrocatalysts for the production of clean and sustainable energy. Notable alternatives in this field are summarized in Table 5 and include hydrogen energy, the electrochemical reduction of CO_2_ into valuable fuels, and the chemical reaction of fuel and oxygen without any by-product [148,149,150,151]. These approaches have emerged as energy strategic solutions to tackle energy supply challenges, promote energy security, and contribute to environmental preservation [152]. Nevertheless, the non-modified and non-activated state of these chars exhibit limited effectiveness in energy storage and green energy applications due to some physicochemical and electrical properties, such as low surface area, inadequate pore features, and suboptimal density and conductivity [153,154]. To overcome this drawback, surface modification and activations are employed to refine the surface characteristics and optimize the material’s functionalities [155,156,157,158].

Water splitting stands as a highly promising method for generating hydrogen energy. The essential process of the electrocatalytic breakage of water into hydrogen and oxygen to harness electric energy and store it within chemical bonds is universally recognized as a critical advancement in sustainable energy conversion and storage systems. Equations (1) and (2) summarize half-reactions of water electrolysis in alkaline conditions, anodic oxygen evolution reaction, and cathodic hydrogen evolution reaction (HER), respectively [163,164]. Once generated, hydrogen serves diverse purposes, playing a substantial role in advancing sustainable energy solutions, including applications in combustion engines, fuel cells for electricity generation, rechargeable metal-air batteries, and large-scale storage within subterranean reservoirs [164,165]. Additionally, efficient electrocatalytic transformation of H_2_ and O_2_ into water serves as a pivotal process in various energy systems. In these processes, the reduction of O_2_ (oxygen reduction reaction, ORR, Equation (3)), frequently aligns with the oxidation of a fuel such as H_2_ (hydrogen oxidation reaction, HOR, Equation (4)), yielding an electromotive force that can energize electronic devices, households, or vehicles. As these processes convert chemical energy into electrical energy, it becomes crucial for electrocatalysis to occur with remarkable rapidity, precision, and energy efficiency [166]. However, conventional electrocatalysts encounter challenges such as high overpotential associated with the HER at the cathode and the OER at the anode; therefore, the efficiency remains hindered [148,166]. Noble metals and their oxides, such as RuO_2_ and IrO_2_, have traditionally served as electrocatalysts, but are limited by their exorbitant cost and instability in alkaline environments [167]. To address these limitations, the doping of carbonaceous materials with heteroatoms such as N and/or S offers a highly efficient approach to decrease overpotential and enhance hydrogen production efficiency, as was reported by, among others, Yuan et al. [168] and Wan et al. [167]. In a study conducted by Yang et al. [148], an environmentally friendly and cost-effective approach to efficient electrocatalysts for hydrogen energy through water splitting was introduced. The researchers synthesized a nanocomposite by growing cobalt oxide nanoparticles on the surface of biochar derived from watermelon peels, followed by activation with KOH for the purpose of electrocatalytic hydrogen production. The authors reported outstanding electrocatalytic performance of the nanocomposite. The OER exhibited a remarkable overpotential of just 237 mV, surpassing commercial RuO_2_. Hydrogen production efficiency was notable due to a low Tafel slope (69.8 mV/dec) and excellent stability over 20 h. In the HER, the reported nanocomposite overpotential (111 mV) exceeded Pt/C (73 mV), but outperformed many carbon-based nanomaterials. Finally, water splitting voltage (1.54 V) was reported to be superior to commercial RuO_2_//Pt/C (1.62 V). Panganoron et al. [151] investigated the potential of repurposing waste biomass as a carbon substrate to augment the performance of α-MnO_2_ catalysts in ORR. Various hydrochars were produced from diverse lignocellulosic residues (corncobs, coffee waste grounds, rice hulls, and coconut lumber sawdust) through HTC under varying temperature and duration conditions, followed by KOH activation and subsequent heat treatment. Electrochemical assessments of ORR activity indicated that the hydrochar/α-MnO_2_ composite produced by corncobs at 250 °C for 12 h exhibited the most favorable outcomes, featuring the highest limiting current density (2.85 mA/cm^2^) and the lowest overpotential (0.50 V) at the onset of ORR among the synthesized composite catalysts. These synthesis conditions preserved oxygen functional groups and fostered the development of porous structures within the corncobs, rendering the catalyst highly stable. However, these values still fell short in comparison to traditional catalysts. This disparity in limiting current density could be attributed to a reduced catalyst loading ratio of α-MnO_2_ to the hydrochar in the synthesis process. Consequently, this work introduces a novel, cost-effective approach to source carbon support from biomass, which can enhance the ORR activity of α-MnO_2_.

Anode, OER:   4OH^−^ → 2H_2_O + O_2_ + 4e^−^ (1)

Cathode, HER:   2H_2_O^−^ + 2e^−^ → 2H_2_ + 2OH^−^ (2)

Cathode, ORR   O_2_ + 4e^−^ + 4H^+^ → 2H_2_O (3)

Anode, HOR   H_2_ + 2OH^−^ → 2H_2_O + 2 e^−^ (4)

Furthermore, given the urgent imperative to address the finite nature of fossil fuel resources and the adverse environmental consequences stemming from fuel combustion, there is an increasing demand for innovative energy storage materials and the urgent reduction of atmospheric CO_2_ [149,155]. Energy storage materials require exceptional electrochemical performance, cost-effectiveness, and scalability. Supercapacitors, also referred to as electrochemical capacitors or ultracapacitors, hold great promise as a new type of energy storage device, given their diverse range of uses from portable batteries to electric vehicles [169]. The electrode materials in supercapacitors are crucial in determining the efficiency and reliability of energy storage systems [22]. Using carbonaceous materials obtained from biomass, such as biochar and hydrochar, has arisen as an attractive approach that promotes circular economy and environmental sustainability and reduces our reliance on finite natural resources [22,159,170]. Tang et al. [159] introduced a straightforward method for producing hierarchical porous carbon materials sourced from waste *Lentinus edodes* by HTC for 24 h and subsequently activated in molten Na_2_CO_3_-K_2_CO_3_ for 1 h. Their study underscores the significant influence of the hydrothermal treatment duration on the resulting carbon materials’ pore size distribution. In a comprehensive evaluation within a three-electrode system employing a 1 M H_2_SO_4_ electrolyte, the hydrochar electrode, when put to the test, displayed remarkable performance attributes. It exhibited an impressive specific capacitance of 389 F/g at a current density of 0.2 A/g, showcasing its excellent capacity. Even under more demanding conditions at 20 A/g, the capacitance remained substantial at 174 F/g, underlining its robust rate capacity. After enduring the rigors of 10,000 cycles at 5 A/g, it retained 90.3% of its initial capacitance, reinforcing its long-lasting performance. A hierarchically porous hydrochar derived from apple pomace was synthesized by [157] into electrode materials for supercapacitors and activated and modified by K_2_FeO_4_. In a three-electrode setup, the modified apple hydrochar demonstrated an impressive specific capacitance of 360.1 F/g at a current density of 0.5 A/g. Moreover, a symmetric supercapacitor composed of two identical apple hydrochar electrodes showcased a remarkable energy density of 16.53 Wh/kg at a designated power density of 250 W/kg, along with 13.17 Wh/kg at 10,000 W/kg. The Coulombic efficiency remained consistently high, ranging from 96% to 100% after 5000 cycles. Impressively, the specific capacitance only declined by 4.3% after 5000 cycles at a current density of 5 A/g, underscoring its exceptional cyclic stability.

The electrochemical transformation of carbon dioxide (CO_2_) into valuable fuels and chemicals is a multifaceted pursuit, with carbon monoxide (CO) considered the most important one as it has high relevance for the chemical industry. This forward-looking endeavor not only presents substantial economic potential, but also aligns seamlessly with environmental objectives by mitigating CO_2_ emissions. It represents a dynamic bridge between economic viability and ecological responsibility [149]. Two novel composites composed of zinc oxide and biochar, one derived from brewed waste coffee powder and the other from chitosan, were reported by Lourenço et al. [149]. These composites were found to be effective catalysts for CO_2_ electrochemical reduction (CO_2_-RR), although the ZnO/Chitosan biochar composite (40.6 wt % of biochar) surpassed the ZnO/coffee biochar composite as a carbon framework for ZnO particles. This superiority was evident through the increased selectivity for CO and the heightened current density achieved at the ZnO/Chitosan electrode (75.6 mA/cm^2^ at −1.3 V vs. RHE). Fu et al. [160] prepared a series of N-doped biochars derived from sugarcane bagasse for evaluation as CO_2_-RR catalysts. The prepared catalyst showcased substantial disparities in their maximum faradaic efficiency concerning CO, ranging from 26.8% to an impressive 94.9% within the voltage range of approximately −0.8 to −0.9 V vs. RHE. Interestingly, it was noted that mere augmentation of the specific surface area and N-doping level of the catalysts did not yield a significant enhancement in the catalytic performance for the CO_2_-RR. A comprehensive analysis, encompassing various factors such as the degree of graphitization, surface hydrophobicity, defect abundance, and optimization of porosity in the N-doped biochar catalyst, effectively boosted its catalytic competence for the CO_2_-RR. The authors revealed a negative correlation between the N-doping content and electrochemical performance. Consequently, the authors underscored the paramount importance of scrutinizing the synergistic impact of diverse properties within N-doped biochar to unravel intricate structure-activity relationships.

Another novel and environmentally friendly application of biochars and hydrochars is their utilization as carbocatalysts in advanced oxidation processes to remove a wide range of pollutants from water and soil, specifically those that are sulfate-based using persulfate (PS), peroxymonosulfate (PMS), or peroxydisulfate (PDS) [88,161,171]. Typically, these techniques rely on metallic catalysts that facilitate radical-induced oxidation of organic substances to achieve a complete mineralization. However, the use of such catalysts often leads to secondary contamination through leaching or sludge generation, which adds extra costs and operational complexities [172]. Carbocatalysts present a promising solution to mitigate the drawbacks associated with conventional metallic catalysts, thereby serving a dual purpose: waste reduction and providing an eco-friendly and cost-effective alternative for water treatment [167]. This approach proves particularly advantageous, considering the typically higher cost of metals, including precious ones, employed as conventional catalysts. Again, most of the studies that have been developed in this field have utilized biochar or hydrochar in an activated form or as composites. This approach allows for the tailoring of their physicochemical characteristics, thereby enhancing their catalytic properties [88,162,173]. Liu and collaborators [161] synthesized catalysts from agri-food residues, including lettuce, taro, and watermelon peel, each with distinct components and tailored functionalities achieved across HTC and a range of temperatures (180–240 °C). These food waste hydrochars were evaluated in the catalytic degradation of the organic pollutant 2,4-dichlorophenoxyacetic acid (2,4-D) initiated by PMS. Notably, HTC of taro, rich in starch, produced at 200 °C, exhibited remarkable catalytic competence, removing 73.5 mg/g of 2,4-D. This exceptional performance was attributed by the authors to the formation of a highly graphitized carbon area characterized by low polarity and an abundance of ketonic functional groups, potentially enhancing the activation of PMS. The activity was ascribed to the contribution of ^•^OH and SO_4_^•−^, O_2_^•−^ presenting a negligible impact on the 2,4-D decomposition in the hydrochar/PMS oxidative system. In a recent study, Qu et al. [162] prepared nitrogen-doped biochar from corn straw loaded with ammonia water and ferrous sulfide via a two-step ball-milling process for PS activation and subsequent phenol degradation. Within the composite/PS system, the degradation of 90% of phenol was influenced by a dynamic interplay involving electron transfer processes and the generation of reactive oxygen species such as SO_4_^•−^, ^•^OH, O^•−^ and ^1^O_2_. The surface-bound S(II) played a dual role: firstly, it interacted with PS, leading to the formation of SO_4_^•−^, and secondly, it expedited the circulation of Fe(III)/Fe(II) by acting as a reducing agent for Fe(III). The system exhibited remarkable versatility, operating effectively across a broad pH spectrum from 3 to 9, displaying strong resistance to interference from coexisting substances, and proving efficient in diverse water compositions. Additionally, the degradation pathways of phenol were elucidated through DFT calculations, revealing intermediate products with reduced toxicity, thus highlighting the significant potential of this innovative approach.

## 5. Conclusions and Future Perspectives

This review provides an assessment of recent advancements in the valorization of agri-food residues in the form of biochar and hydrochar. It explores their utilization as adsorbents in wastewater treatment and examines their potential as promising alternatives in various environmental and energy-related applications. One of the main conclusions is that, through the customization of engineering techniques and the optimization of production conditions, these char materials can acquire a versatile porous structure, remarkable electrochemical properties, and an increased surface area. Their use as an adsorbent is well-established, and there is a clear trend towards engineering adsorbents to overcome the barriers to their future industrial application. These encompass catalytic degradation, CO_2_ fixation, green energy production, and advanced energy storage. Their potential to outperform traditional metal-based catalysts in these domains is particularly noteworthy. Hence, this eco-friendly and sustainable approach to utilizing agri-food residues is in perfect harmony with the principles of Zero Waste and within the framework of a circular economy.

Looking forward, it is crucial to explore the potential future directions in the field of valorizing food manufacturing or agri-food residues through biochar and hydrochar for environmental applications. The rapid progressions and encouraging findings thus far have stablished a strong basis for further research and development. In this section, we aim to provide insights into the exciting possibilities and transformative potential that lie ahead:

- The environmental impact of biochar and hydrochar, including the potential release of toxic compounds, requires further investigation to fully understand its effects when applied in environmental remediation.
- Future research should focus on scaling up the production of biochar and hydrochar, either pristine, activated, or as composites, and evaluating their performance in real-world scenarios, including real wastewaters. This will provide valuable insights into their practical applicability, cost-effectiveness, and environmental implications.
- Despite the potential benefits, research is scarce in the field of incorporating functionalized components and composites with hydrochar. Thus, it creates an exciting opportunity for further investigation and develop functionalized and composited materials with hydrochar.
- Exploring synergistic combinations of different functional groups and materials holds promise for developing biochar composites with improved adsorption capabilities. This could involve integrating biochar and hydrochar with carbonaceous materials, nanomaterials, or polymers to create hybrid composites with enhanced properties, such as increased surface area, porosity, and selectivity for specific pollutants.
- Techno-economic analysis of food waste processing at the pilot and industrial scale is crucial. Therefore, more emphasis should be placed on the economic analysis of char production and the fabrication process for its diverse uses.
- The design of equipment for using the developed chars as remediating materials at the industrial scale should also be a priority research line in the following years in order to validate the postulate processes.
- Conducting a comprehensive life cycle analysis that integrates the production of food manufacturing waste chars, adsorbents or electrode fabrication, and the working systems is necessary. Additionally, there should be a focus on the reuse, stability, and post-treatment of char-based materials.

## Figures and Tables

**Table 1:** Average lignocellulosic composition of some examples of agri-food residues. It should be noted that when “-” appears in the table, it indicates that the data was unavailable.

Agri-Food Residues	Cellulose (%)	Standard Deviation	Hemicellulose (%)	Standard Deviation	Lignin (%)	Standard Deviation	References
Apple pomace	39.25	±4.60	14.90	±5.52	21.35	±3.32	[25]
Banana peels	27.75	±22.98	21.95	±5.02	8.93	±1.24	[25]
Cocoa pod husk	22.90	±4.53	10.75	±2.90	21.00	±9.90	[26]
Grape pomace	10.50	-	6.10	-	31.90	-	[27]
Groundnut shell	35.70	-	18.70	-	30.20	-	[28]
Kiwi peel	37.98	±21.15	-	-	21.37	±5.92	[29]
Olive pomace	23.47	±8.92	29.70	±12.60	38.82	±10.35	[20]
Orange peel	37.08	-	11.04	-	7.52	-	[21]
Pea peel	30.00	-	-	-	25.00	-	[30]
Potato peel	55.00	-	12.00	-	14.00	-	[31]
Rice husk	38.10	±4.24	27.95	±14.64	22.70	±5.52	[25]
Spent coffee grounds	22.65	-	22.65	-	23.90	-	[25,32]
Tomato pomace	7.66	-	7.51	-	37.34	-	[33]
Watermelon rinds	20.00	-	23.00	-	10.00	-	[34]
Wheat straw	40.00	±7.07	25.00	±7.07	15.00	-	[35]

**Table 2:** Elemental composition (carbon (C), hydrogen (H), and oxygen (O)) and ash content of some examples of biochar and hydrochar produced form agri-food residues, as well as process temperature and yield.

Agri-Food Residues	Char	T (°C)	Yield (%)	Ash (%)	C (%)	H (%)	O (%)	Reference
Corncob	Biochar	500	18.9	13.3	77.6	3.05	5.11	[41]
Orange peel	Biochar	300	37.2	1.6	69.3	4.51	22.2	[41]
Wheat straw	Biochar	525	-	9.2	74.4	2.83	-	[41]
Poplar wood	Hydrochar	180	89.9	2.2	50.9	-	-	[46]
Olive waste	Hydrochar	180	75.4	2.2	57.9	-	-	[46]
Wheat straw	Hydrochar	180	80.1	5.8	49.5	-	-	[46]

**Table 4:** Summary of activation strategies for enhanced agri-food residues-based biochar and hydrochar adsorption. The values in the column represent enhancements in adsorption capacity, expressed as multiples (e.g., 6×), compared to its unmodified counterpart. Modification pathway follows the classification given in Figure 9.

Adsorbent	Modification Pathway	Activation Method	Pollutant	Uptake (mg/g)	Adsorption Enhancement	Reference
Loquat cores hydrochar	one-step	H_3_PO_4_, HCl, Citric acid	Diclofenac, prednisolone, antipyrine	-	9.4×	[52]
Shrimp shells hydrochar	pre- post-	NaOH Acetic acid	Methyl orange	755.08	6×	[71]
Orange peel hydrochar	post-	HNO_3_	Methylene blue	115	1.9×	[72]
Potato peel biochar	one-step	KOH	Azorubine	2521	-	[73]
Potato peel biochar	one-step	KOH	Methylene blue	667	-	[73]
Grape pomace hydrochar	post-	KOH	Pb^2+^	137	4.9×	[74]
Apple pomace biochar	pre-	KMnO_4_	Cu^2+^	27.52	-	[75]
Apple pomace biochar	pre-	KMnO_4_	Cd^2+^	30.76	-	[75]
Apple pomace biochar	pre-	KMnO_4_	Zn^2+^	19.69	-	[75]
Apple pomace biochar	pre-	KMnO_4_	Pb^2+^	32.88	-	[75]
Coffee waste biochar	post-	NaOH/H_2_O_2_	Radioactive Sr^2+^	12.71	2.15×	[76]
Corn stalk hydrochar	post-	H_2_O_2_	Cu^2+^	-	2.34×	[77]
Potato peel biochar	post-	steam	Cibacron blue	270.3	2.7×	[78]
Olive stone hydrochar	post-	Air	Fluoxetine	44.1	-	[79]
Olive stone hydrochar	post-	CO_2_	Nicotinic acid	91.9	-	[79]
Corn straw biochar	one-step	NH_3_	Acid orange 7	292	15–20×	[80]
Corn straw biochar	one-step	NH_3_	Methyl blue	436	15–20×	[80]
Corn straw Biochar	one-step	NH_3_	Cu^2+^	103.57	4×	[81]
Corn straw biochar	one-step	NH_3_	Cd^2+^	197.82	4×	[81]
Crayfish shell biochar	post-	Ball-milling	Tetracycline	60.5	1.5×	[82]
Wheat stalk biochar	post-	Ball-milling	Tetracycline	84.54	3×	[83]
Wheat straw biochar	post-	Ball-milling	Volatile organic compounds	11.62–102.22	1.96–3.97×	[84]

**Table 5:** Summary of novel environmental applications of biochar and hydrochar.

Agri-Food Residue	Modification	Application	References
Watermelon peels Biochar	Cobalt oxide nanoparticles	Water splitting catalyst	[148]
Corncobs hydrochar	KOH	ORR catalyst	[151]
*Lentinus edodes* hydrochar	Na_2_CO_3_-K_2_CO_3_	Capacitor	[159]
Apple pomace hydrochar	Ball-milling with K_2_FeO_4_	Supercapacitor	[157]
Brewed waste coffee powder	ZnO	CO_2_-RR catalyst	[149]
Sugarcane bagasse biochar	-	CO_2_-RR catalyst	[160]
Taro hydrochar	-	PMS catalyst	[161]
Corn straw biochar	Two-step ball-milling: ammonia water and ferrous sulfide	PS catalyst	[162]

## Data Availability

The data used to support the findings of this study can be made available by the corresponding author upon request.
